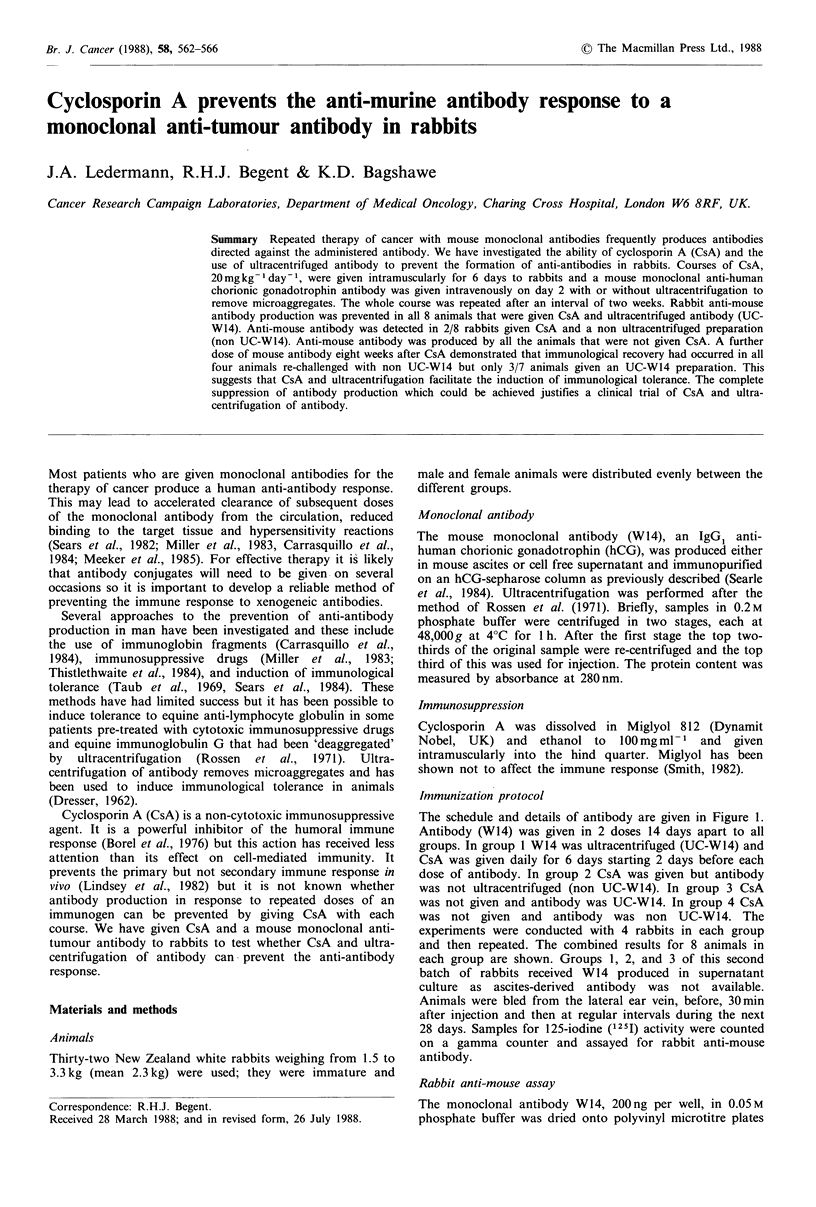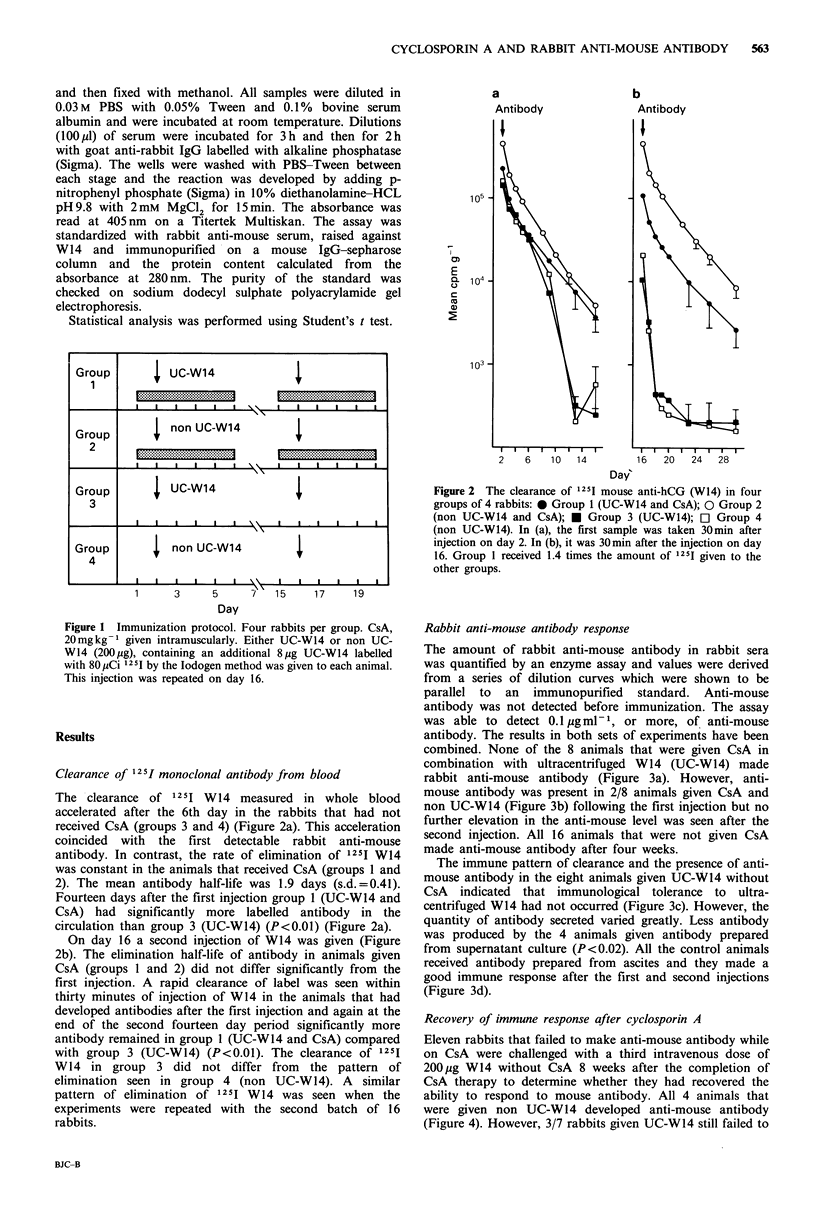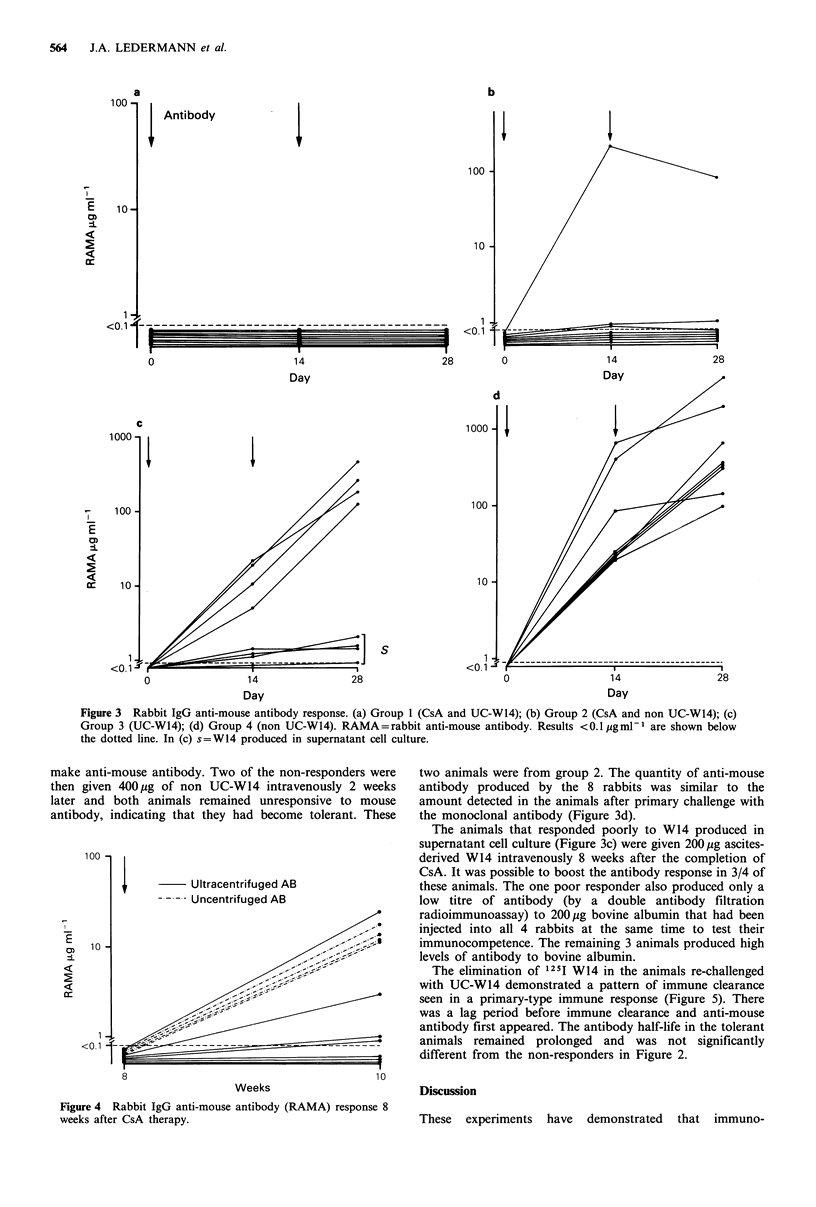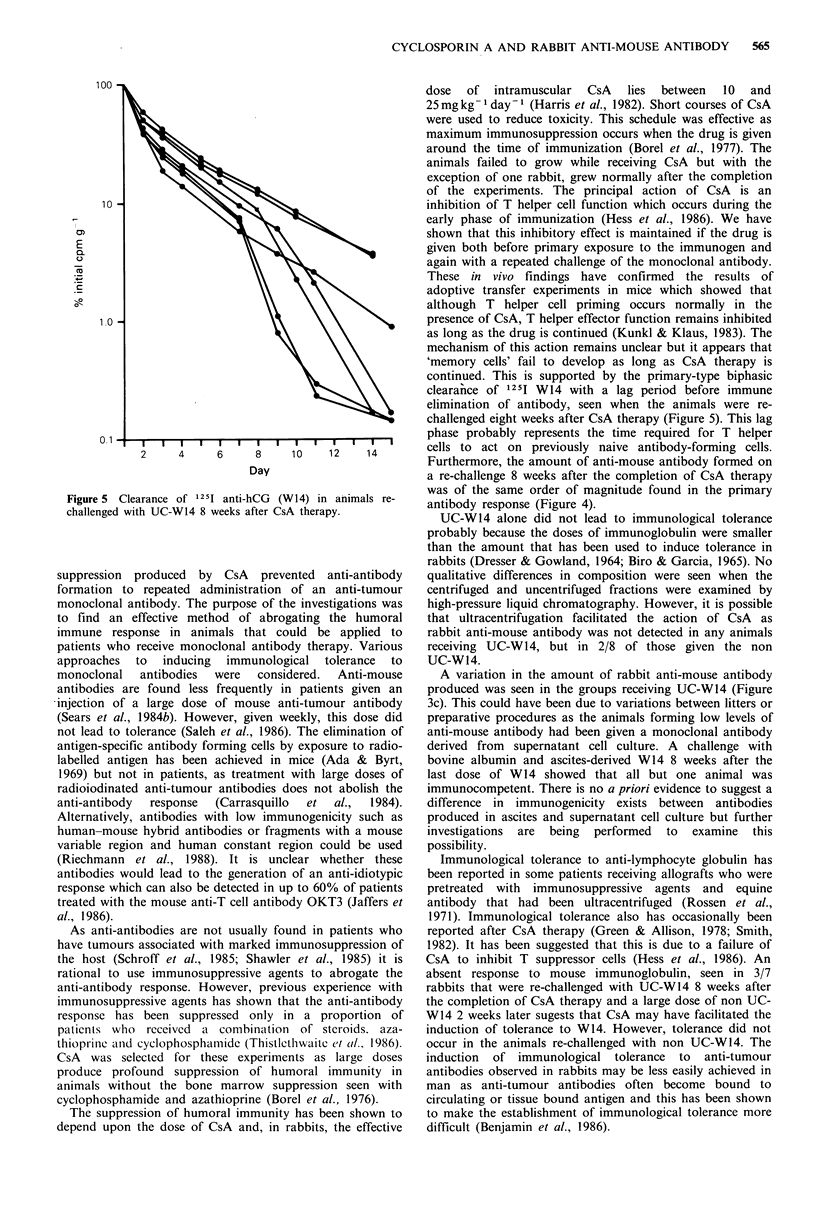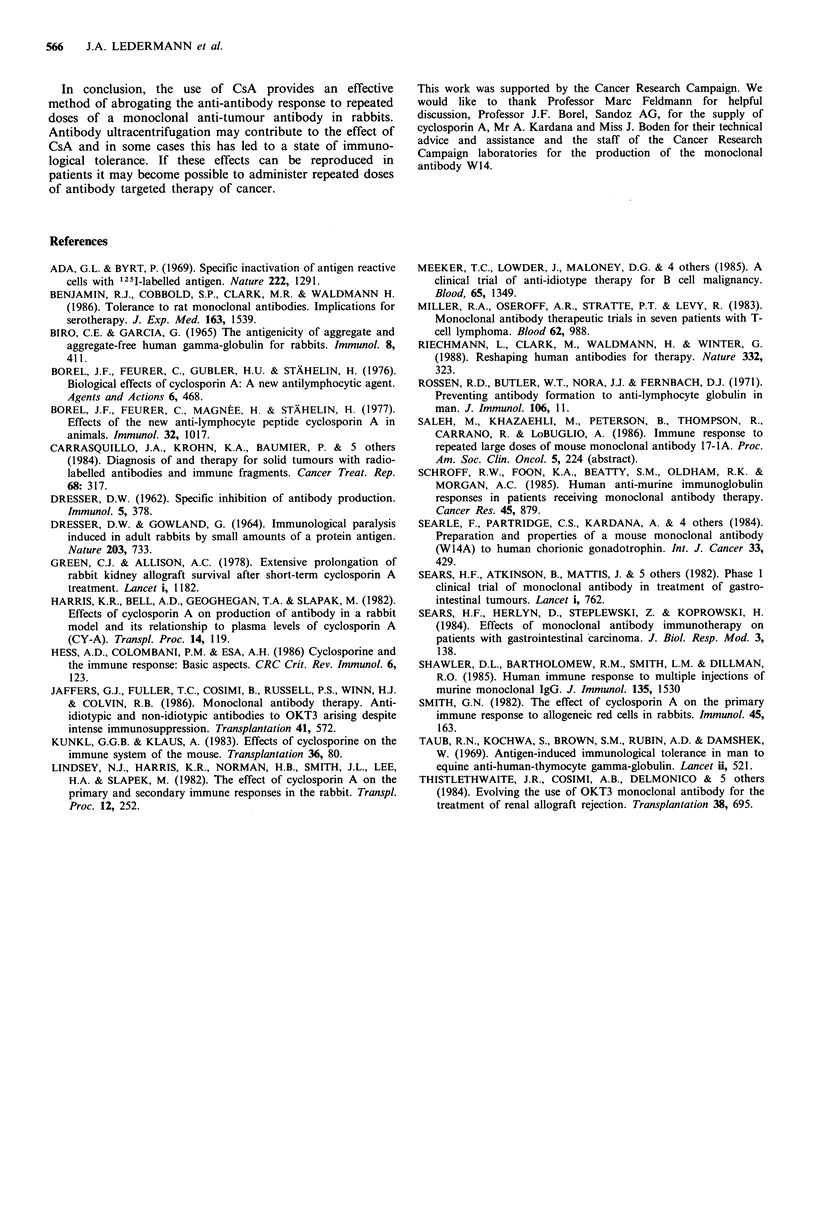# Cyclosporin A prevents the anti-murine antibody response to a monoclonal anti-tumour antibody in rabbits.

**DOI:** 10.1038/bjc.1988.259

**Published:** 1988-11

**Authors:** J. A. Ledermann, R. H. Begent, K. D. Bagshawe

**Affiliations:** Department of Medical Oncology, Charing Cross Hospital, London, UK.

## Abstract

Repeated therapy of cancer with mouse monoclonal antibodies frequently produces antibodies directed against the administered antibody. We have investigated the ability of cyclosporin A (CsA) and the use of ultracentrifuged antibody to prevent the formation of anti-antibodies in rabbits. Courses of CsA, 20 mgkg-1 day-1, were given intramuscularly for 6 days to rabbits and a mouse monoclonal anti-human chorionic gonadotrophin antibody was given intravenously on day 2 with or without ultracentrifugation to remove microaggregates. The whole course was repeated after an interval of two weeks. Rabbit anti-mouse antibody production was prevented in all 8 animals that were given CsA and ultracentrifuged preparation (non UC-W14). Anti-mouse antibody was produced by all the animals that were not given CsA. A further dose of mouse antibody eight weeks after CsA demonstrated that immunological recovery had occurred in all four animals re-challenged with non UC-W14 but only 3/7 animals given an UC-W14 preparation. This suggests that CsA and ultracentrifugation facilitate the induction of immunological tolerance. The complete suppression of antibody production which could be achieved justifies a clinical trial of CsA and ultracentrifugation of antibody.


*Br. J. Cancer* (1988), **58**, 562–566 © The Macmillan Press Ltd., 1988

### Cyclosporin A prevents the anti-murine antibody response to a monoclonal anti-tumour antibody in rabbits

J.A. Ledermann, R.H.J. Begent & K.D. Bagshawe

*Cancer Research Campaign Laboratories, Department of Medical Oncology, Charing Cross Hospital, London W6 8RF, UK.*

**Summary** Repeated therapy of cancer with mouse monoclonal antibodies frequently produces antibodies directed against the administered antibody. We have investigated the ability of cyclosporin A (CsA) and the use of ultracentrifuged antibody to prevent the formation of anti-antibodies in rabbits. Courses of CsA, $20\,mg\,kg^{-1}\,day^{-1}$, were given intramuscularly for 6 days to rabbits and a mouse monoclonal anti-human chorionic gonadotrophin antibody was given intravenously on day 2 with or without ultracentrifugation to remove microaggregates. The whole course was repeated after an interval of two weeks. Rabbit anti-mouse antibody production was prevented in all 8 animals that were given CsA and ultracentrifuged antibody (UC-W14). Anti-mouse antibody was detected in 2/8 rabbits given CsA and a non ultracentrifuged preparation (non UC-W14). Anti-mouse antibody was produced by all the animals that were not given CsA. A further dose of mouse antibody eight weeks after CsA demonstrated that immunological recovery had occurred in all four animals re-challenged with non UC-W14 but only 3/7 animals given an UC-W14 preparation. This suggests that CsA and ultracentrifugation facilitate the induction of immunological tolerance. The complete suppression of antibody production which could be achieved justifies a clinical trial of CsA and ultra-centrifugation of antibody.

Most patients who are given monoclonal antibodies for the therapy of cancer produce a human anti-antibody response. This may lead to accelerated clearance of subsequent doses of the monoclonal antibody from the circulation, reduced binding to the target tissue and hypersensitivity reactions (Sears *et al.*, 1982; Miller *et al.*, 1983, Carrasquillo *et al.*, 1984; Meeker *et al.*, 1985). For effective therapy it is likely that antibody conjugates will need to be given on several occasions so it is important to develop a reliable method of preventing the immune response to xenogeneic antibodies.

Several approaches to the prevention of anti-antibody production in man have been investigated and these include the use of immunoglobin fragments (Carrasquillo *et al.*, 1984), immunosuppressive drugs (Miller *et al.*, 1983; Thistlethwaite *et al.*, 1984), and induction of immunological tolerance (Taub *et al.*, 1969, Sears *et al.*, 1984). These methods have had limited success but it has been possible to induce tolerance to equine anti-lymphocyte globulin in some patients pre-treated with cytotoxic immunosuppressive drugs and equine immunoglobulin G that had been 'deaggregated' by ultracentrifugation (Rossen *et al.*, 1971). Ultra-centrifugation of antibody removes microaggregates and has been used to induce immunological tolerance in animals (Dresser, 1962).

Cyclosporin A (CsA) is a non-cytotoxic immunosuppressive agent. It is a powerful inhibitor of the humoral immune response (Borel *et al.*, 1976) but this action has received less attention than its effect on cell-mediated immunity. It prevents the primary but not secondary immune response *in vivo* (Lindsey *et al.*, 1982) but it is not known whether antibody production in response to repeated doses of an immunogen can be prevented by giving CsA with each course. We have given CsA and a mouse monoclonal anti-tumour antibody to rabbits to test whether CsA and ultra-centrifugation of antibody can prevent the anti-antibody response.

### Materials and methods

### *Animals*

Thirty-two New Zealand white rabbits weighing from 1.5 to 3.3 kg (mean 2.3 kg) were used; they were immature and male and female animals were distributed evenly between the different groups.

### *Monoclonal antibody*

The mouse monoclonal antibody (W14), an $IgG_1$ anti-human chorionic gonadotrophin (hCG), was produced either in mouse ascites or cell free supernatant and immunopurified on an hCG-sepharose column as previously described (Searle *et al.*, 1984). Ultracentrifugation was performed after the method of Rossen *et al.* (1971). Briefly, samples in 0.2 M phosphate buffer were centrifuged in two stages, each at 48,000 *g* at 4°C for 1 h. After the first stage the top two-thirds of the original sample were re-centrifuged and the top third of this was used for injection. The protein content was measured by absorbance at 280 nm.

### *Immunosuppression*

Cyclosporin A was dissolved in Miglyol 812 (Dynamit Nobel, UK) and ethanol to $100\,mg\,ml^{-1}$ and given intramuscularly into the hind quarter. Miglyol has been shown not to affect the immune response (Smith, 1982).

### *Immunization protocol*

The schedule and details of antibody are given in Figure 1. Antibody (W14) was given in 2 doses 14 days apart to all groups. In group 1 W14 was ultracentrifuged (UC-W14) and CsA was given daily for 6 days starting 2 days before each dose of antibody. In group 2 CsA was given but antibody was not ultracentrifuged (non UC-W14). In group 3 CsA was not given and antibody was UC-W14. In group 4 CsA was not given and antibody was non UC-W14. The experiments were conducted with 4 rabbits in each group and then repeated. The combined results for 8 animals in each group are shown. Groups 1, 2, and 3 of this second batch of rabbits received W14 produced in supernatant culture as ascites-derived antibody was not available. Animals were bled from the lateral ear vein, before, 30 min after injection and then at regular intervals during the next 28 days. Samples for 125-iodine ($^{125}I$) activity were counted on a gamma counter and assayed for rabbit anti-mouse antibody.

### *Rabbit anti-mouse assay*

The monoclonal antibody W14, 200 ng per well, in 0.05 M phosphate buffer was dried onto polyvinyl microtitre plates

Correspondence: R.H.J. Begent.
Received 28 March 1988; and in revised form, 26 July 1988.

and then fixed with methanol. All samples were diluted in 0.03 M PBS with 0.05% Tween and 0.1% bovine serum albumin and were incubated at room temperature. Dilutions (100 $\mu$l) of serum were incubated for 3 h and then for 2 h with goat anti-rabbit IgG labelled with alkaline phosphatase (Sigma). The wells were washed with PBS–Tween between each stage and the reaction was developed by adding p-nitrophenyl phosphate (Sigma) in 10% diethanolamine–HCL pH 9.8 with 2 mM $MgCl_2$ for 15 min. The absorbance was read at 405 nm on a Titertek Multiskan. The assay was standardized with rabbit anti-mouse serum, raised against W14 and immunopurified on a mouse IgG–sepharose column and the protein content calculated from the absorbance at 280 nm. The purity of the standard was checked on sodium dodecyl sulphate polyacrylamide gel electrophoresis.

Statistical analysis was performed using Student's $t$ test.

**Figure 1** Immunization protocol. Four rabbits per group. CsA, 20 mg kg$^{-1}$ given intramuscularly. Either UC-W14 or non UC-W14 (200 $\mu$g), containing an additional 8 $\mu$g UC-W14 labelled with 80 $\mu$Ci $^{125}$I by the Iodogen method was given to each animal. This injection was repeated on day 16.

**Figure 2** The clearance of $^{125}$I mouse anti-hCG (W14) in four groups of 4 rabbits: ● Group 1 (UC-W14 and CsA); ○ Group 2 (non UC-W14 and CsA); ■ Group 3 (UC-W14); □ Group 4 (non UC-W14). In (a), the first sample was taken 30 min after injection on day 2. In (b), it was 30 min after the injection on day 16. Group 1 received 1.4 times the amount of $^{125}$I given to the other groups.

### Results

### *Clearance of $^{125}$I monoclonal antibody from blood*

The clearance of $^{125}$I W14 measured in whole blood accelerated after the 6th day in the rabbits that had not received CsA (groups 3 and 4) (Figure 2a). This acceleration coincided with the first detectable rabbit anti-mouse antibody. In contrast, the rate of elimination of $^{125}$I W14 was constant in the animals that received CsA (groups 1 and 2). The mean antibody half-life was 1.9 days (s.d. = 0.41). Fourteen days after the first injection group 1 (UC-W14 and CsA) had significantly more labelled antibody in the circulation than group 3 (UC-W14) ($P$<0.01) (Figure 2a).

On day 16 a second injection of W14 was given (Figure 2b). The elimination half-life of antibody in animals given CsA (groups 1 and 2) did not differ significantly from the first injection. A rapid clearance of label was seen within thirty minutes of injection of W14 in the animals that had developed antibodies after the first injection and again at the end of the second fourteen day period significantly more antibody remained in group 1 (UC-W14 and CsA) compared with group 3 (UC-W14) ($P$<0.01). The clearance of $^{125}$I W14 in group 3 did not differ from the pattern of elimination seen in group 4 (non UC-W14). A similar pattern of elimination of $^{125}$I W14 was seen when the experiments were repeated with the second batch of 16 rabbits.

### *Rabbit anti-mouse antibody response*

The amount of rabbit anti-mouse antibody in rabbit sera was quantified by an enzyme assay and values were derived from a series of dilution curves which were shown to be parallel to an immunopurified standard. Anti-mouse antibody was not detected before immunization. The assay was able to detect 0.1 $\mu$g ml$^{-1}$, or more, of anti-mouse antibody. The results in both sets of experiments have been combined. None of the 8 animals that were given CsA in combination with ultracentrifuged W14 (UC-W14) made rabbit anti-mouse antibody (Figure 3a). However, anti-mouse antibody was present in 2/8 animals given CsA and non UC-W14 (Figure 3b) following the first injection but no further elevation in the anti-mouse level was seen after the second injection. All 16 animals that were not given CsA made anti-mouse antibody after four weeks.

The immune pattern of clearance and the presence of anti-mouse antibody in the eight animals given UC-W14 without CsA indicated that immunological tolerance to ultra-centrifuged W14 had not occurred (Figure 3c). However, the quantity of antibody secreted varied greatly. Less antibody was produced by the 4 animals given antibody prepared from supernatant culture ($P$<0.02). All the control animals received antibody prepared from ascites and they made a good immune response after the first and second injections (Figure 3d).

### *Recovery of immune response after cyclosporin A*

Eleven rabbits that failed to make anti-mouse antibody while on CsA were challenged with a third intravenous dose of 200 $\mu$g W14 without CsA 8 weeks after the completion of CsA therapy to determine whether they had recovered the ability to respond to mouse antibody. All 4 animals that were given non UC-W14 developed anti-mouse antibody (Figure 4). However, 3/7 rabbits given UC-W14 still failed to

**Figure 3** Rabbit IgG anti-mouse antibody response. (a) Group 1 (CsA and UC-W14); (b) Group 2 (CsA and non UC-W14); (c) Group 3 (UC-W14); (d) Group 4 (non UC-W14). RAMA = rabbit anti-mouse antibody. Results $<0.1\,\mu g\,ml^{-1}$ are shown below the dotted line. In (c) $s$ = W14 produced in supernatant cell culture.

make anti-mouse antibody. Two of the non-responders were then given 400 μg of non UC-W14 intravenously 2 weeks later and both animals remained unresponsive to mouse antibody, indicating that they had become tolerant. These two animals were from group 2. The quantity of anti-mouse antibody produced by the 8 rabbits was similar to the amount detected in the animals after primary challenge with the monoclonal antibody (Figure 3d).

**Figure 4** Rabbit IgG anti-mouse antibody (RAMA) response 8 weeks after CsA therapy.

The animals that responded poorly to W14 produced in supernatant cell culture (Figure 3c) were given 200 μg ascites-derived W14 intravenously 8 weeks after the completion of CsA. It was possible to boost the antibody response in 3/4 of these animals. The one poor responder also produced only a low titre of antibody (by a double antibody filtration radioimmunoassay) to 200 μg bovine albumin that had been injected into all 4 rabbits at the same time to test their immunocompetence. The remaining 3 animals produced high levels of antibody to bovine albumin.

The elimination of $^{125}I$ W14 in the animals re-challenged with UC-W14 demonstrated a pattern of immune clearance seen in a primary-type immune response (Figure 5). There was a lag period before immune clearance and anti-mouse antibody first appeared. The antibody half-life in the tolerant animals remained prolonged and was not significantly different from the non-responders in Figure 2.

### Discussion

These experiments have demonstrated that immuno-

**Figure 5** Clearance of $^{125}$I anti-hCG (W14) in animals re-challenged with UC-W14 8 weeks after CsA therapy.

suppression produced by CsA prevented anti-antibody formation to repeated administration of an anti-tumour monoclonal antibody. The purpose of the investigations was to find an effective method of abrogating the humoral immune response in animals that could be applied to patients who receive monoclonal antibody therapy. Various approaches to inducing immunological tolerance to monoclonal antibodies were considered. Anti-mouse antibodies are found less frequently in patients given an injection of a large dose of mouse anti-tumour antibody (Sears *et al.*, 1984*b*). However, given weekly, this dose did not lead to tolerance (Saleh *et al.*, 1986). The elimination of antigen-specific antibody forming cells by exposure to radio-labelled antigen has been achieved in mice (Ada & Byrt, 1969) but not in patients, as treatment with large doses of radioiodinated anti-tumour antibodies does not abolish the anti-antibody response (Carrasquillo *et al.*, 1984). Alternatively, antibodies with low immunogenicity such as human–mouse hybrid antibodies or fragments with a mouse variable region and human constant region could be used (Riechmann *et al.*, 1988). It is unclear whether these antibodies would lead to the generation of an anti-idiotypic response which can also be detected in up to 60% of patients treated with the mouse anti-T cell antibody OKT3 (Jaffers *et al.*, 1986).

As anti-antibodies are not usually found in patients who have tumours associated with marked immunosuppression of the host (Schroff *et al.*, 1985; Shawler *et al.*, 1985) it is rational to use immunosuppressive agents to abrogate the anti-antibody response. However, previous experience with immunosuppressive agents has shown that the anti-antibody response has been suppressed only in a proportion of patients who received a combination of steroids, azathioprine and cyclophosphamide (Thistlethwaite *et al.*, 1986). CsA was selected for these experiments as large doses produce profound suppression of humoral immunity in animals without the bone marrow suppression seen with cyclophosphamide and azathioprine (Borel *et al.*, 1976).

The suppression of humoral immunity has been shown to depend upon the dose of CsA and, in rabbits, the effective dose of intramuscular CsA lies between 10 and 25 mg kg$^{-1}$ day$^{-1}$ (Harris *et al.*, 1982). Short courses of CsA were used to reduce toxicity. This schedule was effective as maximum immunosuppression occurs when the drug is given around the time of immunization (Borel *et al.*, 1977). The animals failed to grow while receiving CsA but with the exception of one rabbit, grew normally after the completion of the experiments. The principal action of CsA is an inhibition of T helper cell function which occurs during the early phase of immunization (Hess *et al.*, 1986). We have shown that this inhibitory effect is maintained if the drug is given both before primary exposure to the immunogen and again with a repeated challenge of the monoclonal antibody. These *in vivo* findings have confirmed the results of adoptive transfer experiments in mice which showed that although T helper cell priming occurs normally in the presence of CsA, T helper effector function remains inhibited as long as the drug is continued (Kunkl & Klaus, 1983). The mechanism of this action remains unclear but it appears that 'memory cells' fail to develop as long as CsA therapy is continued. This is supported by the primary-type biphasic clearance of $^{125}$I W14 with a lag period before immune elimination of antibody, seen when the animals were re-challenged eight weeks after CsA therapy (Figure 5). This lag phase probably represents the time required for T helper cells to act on previously naive antibody-forming cells. Furthermore, the amount of anti-mouse antibody formed on a re-challenge 8 weeks after the completion of CsA therapy was of the same order of magnitude found in the primary antibody response (Figure 4).

UC-W14 alone did not lead to immunological tolerance probably because the doses of immunoglobulin were smaller than the amount that has been used to induce tolerance in rabbits (Dresser & Gowland, 1964; Biro & Garcia, 1965). No qualitative differences in composition were seen when the centrifuged and uncentrifuged fractions were examined by high-pressure liquid chromatography. However, it is possible that ultracentrifugation facilitated the action of CsA as rabbit anti-mouse antibody was not detected in any animals receiving UC-W14, but in 2/8 of those given the non UC-W14.

A variation in the amount of rabbit anti-mouse antibody produced was seen in the groups receiving UC-W14 (Figure 3c). This could have been due to variations between litters or preparative procedures as the animals forming low levels of anti-mouse antibody had been given a monoclonal antibody derived from supernatant cell culture. A challenge with bovine albumin and ascites-derived W14 8 weeks after the last dose of W14 showed that all but one animal was immunocompetent. There is no *a priori* evidence to suggest a difference in immunogenicity exists between antibodies produced in ascites and supernatant cell culture but further investigations are being performed to examine this possibility.

Immunological tolerance to anti-lymphocyte globulin has been reported in some patients receiving allografts who were pretreated with immunosuppressive agents and equine antibody that had been ultracentrifuged (Rossen *et al.*, 1971). Immunological tolerance also has occasionally been reported after CsA therapy (Green & Allison, 1978; Smith, 1982). It has been suggested that this is due to a failure of CsA to inhibit T suppressor cells (Hess *et al.*, 1986). An absent response to mouse immunoglobulin, seen in 3/7 rabbits that were re-challenged with UC-W14 8 weeks after the completion of CsA therapy and a large dose of non UC-W14 2 weeks later sugests that CsA may have facilitated the induction of tolerance to W14. However, tolerance did not occur in the animals re-challenged with non UC-W14. The induction of immunological tolerance to anti-tumour antibodies observed in rabbits may be less easily achieved in man as anti-tumour antibodies often become bound to circulating or tissue bound antigen and this has been shown to make the establishment of immunological tolerance more difficult (Benjamin *et al.*, 1986).

In conclusion, the use of CsA provides an effective method of abrogating the anti-antibody response to repeated doses of a monoclonal anti-tumour antibody in rabbits. Antibody ultracentrifugation may contribute to the effect of CsA and in some cases this has led to a state of immunological tolerance. If these effects can be reproduced in patients it may become possible to administer repeated doses of antibody targeted therapy of cancer.

This work was supported by the Cancer Research Campaign. We would like to thank Professor Marc Feldmann for helpful discussion, Professor J.F. Borel, Sandoz AG, for the supply of cyclosporin A, Mr A. Kardana and Miss J. Boden for their technical advice and assistance and the staff of the Cancer Research Campaign laboratories for the production of the monoclonal antibody W14.

### References

ADA, G.L. & BYRT, P. (1969). Specific inactivation of antigen reactive cells with $^{125}$I-labelled antigen. *Nature* **222**, 1291.

BENJAMIN, R.J., COBBOLD, S.P., CLARK, M.R. & WALDMANN H. (1986). Tolerance to rat monoclonal antibodies. Implications for serotherapy. *J. Exp. Med.* **163**, 1539.

BIRO, C.E. & GARCIA, G. (1965) The antigenicity of aggregate and aggregate-free human gamma-globulin for rabbits. *Immunol.* **8**, 411.

BOREL, J.F., FEURER, C., GUBLER, H.U. & STÄHELIN, H. (1976). Biological effects of cyclosporin A: A new antilymphocytic agent. *Agents and Actions* **6**, 468.

BOREL, J.F., FEURER, C., MAGNÉE, H. & STÄHELIN, H. (1977). Effects of the new anti-lymphocyte peptide cyclosporin A in animals. *Immunol.* **32**, 1017.

CARRASQUILLO, J.A., KROHN, K.A., BAUMIER, P. & 5 others (1984). Diagnosis of and therapy for solid tumours with radiolabelled antibodies and immune fragments. *Cancer Treat. Rep.* **68**: 317.

DRESSER, D.W. (1962). Specific inhibition of antibody production. *Immunol.* **5**, 378.

DRESSER, D.W. & GOWLAND, G. (1964). Immunological paralysis induced in adult rabbits by small amounts of a protein antigen. *Nature* **203**, 733.

GREEN, C.J. & ALLISON, A.C. (1978). Extensive prolongation of rabbit kidney allograft survival after short-term cyclosporin A treatment. *Lancet* **i**, 1182.

HARRIS, K.R., BELL, A.D., GEOGHEGAN, T.A. & SLAPAK, M. (1982). Effects of cyclosporin A on production of antibody in a rabbit model and its relationship to plasma levels of cyclosporin A (CY-A). *Transpl. Proc.* **14**, 119.

HESS, A.D., COLOMBANI, P.M. & ESA, A.H. (1986) Cyclosporine and the immune response: Basic aspects. *CRC Crit. Rev. Immunol.* **6**, 123.

JAFFERS, G.J., FULLER, T.C., COSIMI, B., RUSSELL, P.S., WINN, H.J. & COLVIN, R.B. (1986). Monoclonal antibody therapy. Anti-idiotypic and non-idiotypic antibodies to OKT3 arising despite intense immunosuppression. *Transplantation* **41**, 572.

KUNKL, G.G.B. & KLAUS, A. (1983). Effects of cyclosporine on the immune system of the mouse. *Transplantation* **36**, 80.

LINDSEY, N.J., HARRIS, K.R., NORMAN, H.B., SMITH, J.L., LEE, H.A. & SLAPEK, M. (1982). The effect of cyclosporin A on the primary and secondary immune responses in the rabbit. *Transpl. Proc.* **12**, 252.

MEEKER, T.C., LOWDER, J., MALONEY, D.G. & 4 others (1985). A clinical trial of anti-idiotype therapy for B cell malignancy. *Blood*, **65**, 1349.

MILLER, R.A., OSEROFF, A.R., STRATTE, P.T. & LEVY, R. (1983). Monoclonal antibody therapeutic trials in seven patients with T-cell lymphoma. *Blood* **62**, 988.

RIECHMANN, L., CLARK, M., WALDMANN, H. & WINTER, G. (1988). Reshaping human antibodies for therapy. *Nature* **332**, 323.

ROSSEN, R.D., BUTLER, W.T., NORA, J.J. & FERNBACH, D.J. (1971). Preventing antibody formation to anti-lymphocyte globulin in man. *J. Immunol.* **106**, 11.

SALEH, M., KHAZAEHLI, M., PETERSON, B., THOMPSON, R., CARRANO, R. & LoBUGLIO, A. (1986). Immune response to repeated large doses of mouse monoclonal antibody 17-1A. *Proc. Am. Soc. Clin. Oncol.* **5**, 224 (abstract).

SCHROFF, R.W., FOON, K.A., BEATTY, S.M., OLDHAM, R.K. & MORGAN, A.C. (1985). Human anti-murine immunoglobulin responses in patients receiving monoclonal antibody therapy. *Cancer Res.* **45**, 879.

SEARLE, F., PARTRIDGE, C.S., KARDANA, A. & 4 others (1984). Preparation and properties of a mouse monoclonal antibody (W14A) to human chorionic gonadotrophin. *Int. J. Cancer* **33**, 429.

SEARS, H.F., ATKINSON, B., MATTIS, J. & 5 others (1982). Phase 1 clinical trial of monoclonal antibody in treatment of gastrointestinal tumours. *Lancet* **i**, 762.

SEARS, H.F., HERLYN, D., STEPLEWSKI, Z. & KOPROWSKI, H. (1984). Effects of monoclonal antibody immunotherapy on patients with gastrointestinal carcinoma. *J. Biol. Resp. Mod.* **3**, 138.

SHAWLER, D.L., BARTHOLOMEW, R.M., SMITH, L.M. & DILLMAN, R.O. (1985). Human immune response to multiple injections of murine monoclonal IgG. *J. Immunol.* **135**, 1530

SMITH, G.N. (1982). The effect of cyclosporin A on the primary immune response to allogeneic red cells in rabbits. *Immunol.* **45**, 163.

TAUB, R.N., KOCHWA, S., BROWN, S.M., RUBIN, A.D. & DAMSHEK, W. (1969). Antigen-induced immunological tolerance in man to equine anti-human-thymocyte gamma-globulin. *Lancet* **ii**, 521.

THISTLETHWAITE, J.R., COSIMI, A.B., DELMONICO & 5 others (1984). Evolving the use of OKT3 monoclonal antibody for the treatment of renal allograft rejection. *Transplantation* **38**, 695.